# Immunogram defines four cancer-immunity cycle phenotypes with distinct clonal selection patterns across solid tumors

**DOI:** 10.1186/s12967-023-04765-5

**Published:** 2024-01-20

**Authors:** Ying Hu, Huaibo Sun, Wei Shi, Chen Chen, Xueying Wu, Yu Jiang, Guoying Zhang, Na Li, Jin Song, Hao Zhang, Baiyong Shen, Hui Zeng, Henghui Zhang

**Affiliations:** 1 Biomedical Innovation Center, Beijing Shijitan Hospital, Capital Medical University, Beijing, 100038 China; 2 Beijing Key Laboratory for Therapeutic Cancer Vaccines, Beijing Shijitan Hospital, Capital Medical University, Beijing, 100038 China; 3 Beijing SinoMDgene Technology CO., LTD, Beijing, 100176 China; 4 Department of General Surgery, Huashan Hospital, Fudan University, Shanghai, 200040 China; 5 Beijing Immupeutics Medicine Technology Limited, Beijing, 102609 China; 6 Department of General Surgery, Pancreatic Disease Center, Research Institute of Pancreatic Diseases, Ruijin Hospital, Shanghai Jiao Tong University School of Medicine, Shanghai, 200025 China; 7 State Key Laboratory of Oncogenes and Related Genes, National Research Center for Translational Medicine (Shanghai), Shanghai, 200025 China; 8 Beijing Engineering Research Center of Immunocellular therapy, Beijing, 102609 China

**Keywords:** Cancer-immunity cycle, Immunogram, Clonal selection, Cancer evolution, Immune checkpoint inhibitor

## Abstract

**Background:**

The cancer-immunity cycle (CI cycle) provides a theoretical framework to illustrate the process of the anticancer immune response. Recently, the update of the CI cycle theory emphasizes the importance of tumor’s immunological phenotype. However, there is lack of immunological phenotype of pan-cancer based on CI cycle theory.

**Methods:**

Here, we applied a visualizing method termed ‘cancer immunogram’ to visualize the state of CI cycle of 8460 solid tumors from TCGA cohort. Unsupervised clustering of the cancer immunogram was performed using the nonnegative matrix factorization (NMF) analysis. We applied an evolutionary genomics approach (dN/dS ratio) to evaluate the clonal selection patterns of tumors with distinct immunogram subtypes.

**Results:**

We defined four major CI cycle patterns across 32 cancer types using a cancer immunogram approach. Immunogram-I was characterized by ‘hot’ and ‘exhausted’ features, indicating a favorable prognosis. Strikingly, immunogram-II, immunogram-III, and immunogram-IV represented distinct immunosuppressive patterns of ‘cold’ tumor. Immunogram-II was characterized by ‘cold’ and ‘radical’ features, which represented increased expression of immune inhibitor molecules and high levels of positive selection, indicating the worst prognosis. Immunogram-III was characterized by ‘cold’ and ‘recognizable’ features and upregulated expression of MHC I molecules. Immunogram-IV was characterized by ‘cold’ and ‘inert’ features, which represented overall immunosuppression, lower levels of immunoediting and positive selection, and accumulation of more tumor neoantigens. In particular, favorable overall survival was observed in metastatic urothelial cancer patients with immunogram-I and immunogram-IV after immune checkpoint inhibitor (ICI) therapy. Meanwhile, a higher response rate to ICI therapy was observed in metastatic gastric cancer patients with immunogram-I phenotype.

**Conclusions:**

Our findings provide new insight into the interaction between immunity and cancer evolution, which may contribute to optimizing immunotherapy strategies.

**Supplementary Information:**

The online version contains supplementary material available at 10.1186/s12967-023-04765-5.

## Introduction

Immune checkpoint inhibitor (ICI) therapy represents a conceptual revolution in the management of multiple cancer types [1, 2]. However, a durable response to ICI therapy was only achieved in a subset of patients. In most solid tumors, response rates range from 15 to 30% [3]. To improve the durability and effectiveness of antitumor immune response, it is necessary to comprehensively assess the antitumor response status and subsequently optimize the treatment strategy for individual patient. The cancer-immunity cycle theory provides a summary of the understanding of the process of the antitumor immune response [4]. Briefly, the antitumor immune response has been illustrated as seven important steps: cancer cell antigen release, cancer antigen presentation by antigen-presenting cells, priming and activation of the effector T cell response, trafficking of T cells to tumors, infiltration of T cells into tumors, recognition of cancer cells by T cells, and ultimately killing of cancer cells [4].

Recently, the update of the cancer-immunity cycle theory emphasizes the importance of tumor’s immunological phenotype [5]. Tumors within same cancer type can still be characterized by distinct immunological phenotypes. Meanwhile, it is important that several immunotypes also occur in all types of solid tumors, regardless of origin [5]. Therefore, immunotypes of solid tumors will provide valuables framework to deepen our understanding of the mechanistic basis of the response or resistance to therapy and then guide the future development of optimized treatment strategies. Previous studies have proposed different classifications of tumor’s immune subtypes [6, 7]. However, the comprehensive cancer-immunity cycle phenotypes across solid tumors are sill lack of assessment.

Cancer immunograms provide a visualization and clear view of the cancer-immune cycle status of each patient. The advantage of this concept is that it comprehensively integrates omics data and provides visualizing information on an individual patients’ cancer-immunity cycle status for clinical oncologists [5, 6]. Here, we defined four major cancer-immunity cycle patterns across 32 cancer types using a cancer immunogram approach. Immunogram-I was characterized by ‘hot’ tumor features with activated and exhausted immune patterns, indicating a favorable prognosis. Strikingly, we found that immunogram-II, immunogram-III, and immunogram-IV represented distinct immunosuppressive subtypes of ‘cold’ tumors. We also found that immunogram subtypes correlated with patient response to ICI therapy in metastatic urothelial cancer and gastric cancer. These findings may contribute to the understanding of the interaction between cancer and immunity, which may provide a resource to improve anticancer strategies.

## Materials and methods

### Data collection

A total of 8460 patients with solid tumors were enrolled in this study. All the data were collected from publicly available cohorts (TCGA cohort and IMvigor210 cohort) [8, 9]. A total of 8460 patients with solid tumors were enrolled in this study according to the following inclusion criteria: the patients were included in the TCGA pancancer cohort and had available clinical, RNA sequencing and whole exome sequencing (WES) data from a previously published study [8] (https://gdc.cancer.gov/about-data/publications/PanCan-CellOfOrigin). The primary tumors data were selected in this study. The clinical data, RNA sequencing and WES data of the IMvigor210 and metastatic gastric cancer cohort were available from a previously published study respectively [9, 10] (http://research-pub.gene.com/IMvigor210CoreBiologies). LIRI-JP HCC cohorts with WES data, RNA sequencing data and clinical data were downloaded from International Cancer Genome Consortium (ICGC)(https://dcc.icgc.org/). The sample annotation was provided in Table S1–S5 (Additional file 1: Table S1–S5).

### Cancer immunogram analysis and NMF clustering

According to a previous study [11], the steps of the cancer-immunity cycle are described by eight axes according to eight immunogram scores (IGSs). Gene set variation analysis (GSVA) was performed to assess the value of IGS using the GSVA R package. Tumor neoantigen burden (TNB) data of the TCGA cohort were available from a previously published study [7]. As previously reported [11], the steps of the cancer-immunity cycle are described by eight axes of IGSs as follows: IGS1, T cell immunity; IGS2, tumor antigenicity; IGS3, priming and activation; IGS4, trafficking and infiltration; IGS5, recognition of tumor cells; IGS6, inhibitor cells; IGS7, checkpoint expression; and IGS8, inhibitory molecules. The gene sets for IGS1, IGS3, IGS4, IGS5, IGS6, IGS7, and IGS8 were used in a previous study [11]. Gene set variation analysis (GSVA) was performed to assess the value of IGS using the GSVA R package. Unsupervised clustering of the cancer immunogram was performed using the nonnegative matrix factorization (NMF) algorithm, as described in a previous study [12, 13]. NMF clustering was performed with the NMF R package (Version 0.22.0). The standard “brunet” option was selected, and 200 iterations were conducted. The range of cluster numbers (k) was set as 2 to 7. After comparing clustering quality through the NMF R package, the optimal clustering number was identified as 4.

### Clonal selection of cancer evolution

We applied an evolutionary genomics approach (dN/dS ratio) to evaluate the clonal selection patterns of tumors with distinct immunogram subtypes. The dN/dS ratio, the ratio of nonsynonymous mutations to synonymous mutations, is a method for identifying the selection pressure exerted during cancer evolution [14]. This tool is based on the point that synonymous mutations have an evolutionarily neutral mutation background. Therefore, when nonsynonymous mutations are positively selected, the dN/dS ratio is > 1; in contrast, when nonsynonymous mutations are negatively selected, the dN/dS ratio is < 1. Here, we used this method to determine the clonal selection pattern during cancer evolution as previously described [14]. The dN/dS ratio was estimated using the R package available from a previously published study (https://github.com/im3sanger/dndscv).

### Immune and molecular features

The gene sets for immunostimulators, immunoinhibitors, chemokines, and MHC class-I and MHC class-II molecules were described in a previous study [7, 15]. The immune signatures were measured as the geometric mean of gene expression in log2 of transcripts per million (TPM) + 1. The immunoediting score was evaluated as described in a previous study [1]. The TNB score, tumor mutation burden (TMB) score, CNV burden score, LOH score, HRD score, leukocyte fraction, stroma fraction, aneuploidy score, number of TCR clones, and TCR diversity score (Shannon Entropy) of the TCGA cohort were available from a previously published TCGA study [7]. The MATH algorithm was applied as previously described [16]. The relative abundance of 28 immune cell subsets that infiltrated the tumor was available from a previously published TCGA study [7].

### Statistical analysis

Data are expressed as the mean and standard error of the mean (SEM). Group values were assessed using a normal distribution test. For normally distributed data, group means were compared by Student’s t test, and nonparametric tests were used when the data were not normally distributed. Differences with p < 0.05 were defined as statistically significant. A univariable Cox proportional hazards model was applied to evaluate the variables in relation to OS, and multivariable Cox proportional hazards model was to assess the effect of multiple variables on OS. Statistical analysis was performed using R (Version 4.0.2).

## Results

### Pan-cancer samples with the four immunogram subtypes and distinct prognoses

A total of 8460 patients with solid tumors were enrolled in this study according to the following inclusion criteria: the patients were included in the TCGA pan-cancer cohort and had available clinical, RNA sequencing and whole exome sequencing (WES) data from a previously published study [8]. Based on the theory of the cancer-immunity cycle, we adopted a cancer immunogram to illustrate the antitumor immune response across cancers. Cancer immunograms could evaluate and visualize the cancer-immunity cycle status for each patient by eight IGSs: IGS1, T cell immunity; IGS2, tumor neoantigen burden (TNB); IGS3, priming and activation; IGS4, trafficking and infiltration; IGS5, recognition of tumor cells; IGS6, inhibitor cells; IGS7, checkpoint expression; and IGS8, inhibitor molecules [11].

**Fig. 1:**
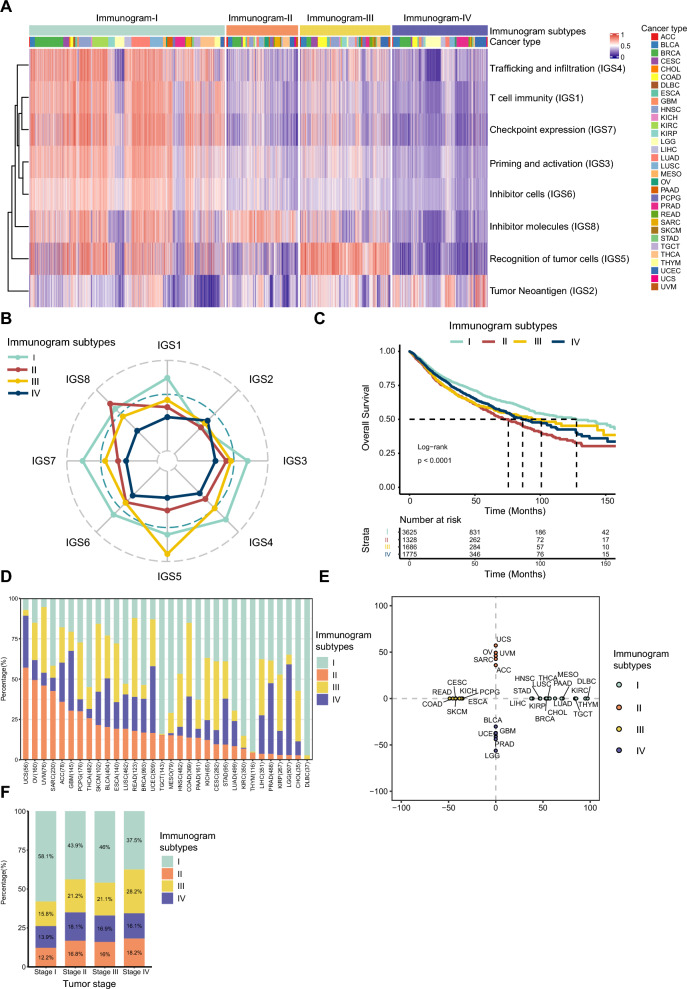
Immunogram subtypes and prognosis in a pan-cancer cohort.** A** NMF clustering analysis of immunograms based on the eight axes of the IGS for 8460 patients in the TCGA cohort. B, The radar plot showed that the immunogram patterns of the four clusters were distinct. The axes of the radar chart were generated according to the median IGS for the four immunogram subtypes. **C** Kaplan–Meier curves for the OS of patients in the TCGA cohort stratified by the four immunogram subtypes. The log-rank test yielded P < 0.0001. **D** The proportion of samples with each immunogram subtype is shown. **E** Distribution of the four immunogram subtypes within TCGA tumors. **F** The proportion of Immunogram-I, II, III, and IV in the progression from stage I to stage IV tumors

We assessed the IGS profiles in 8460 tumors comprising 32 diverse cancer types in TCGA. The immunogram patterns of pan-cancer were separated into four clusters (termed immunogram-I to immunogram-IV, Fig. 1A) by NMF clustering analysis. Immunogram-I was characterized by higher scores for both stimulatory factors (IGS1, IGS3, IGS4, IGS5) and inhibitory factors (IGS6, IGS7, IGS8) of the antitumor immune response, indicating that Immunogram-I represents activated and exhausted immune patterns. In contrast, immunogram-IV showed low scores for both stimulatory and inhibitory factors but a relatively high TNB (IGS2). The immunogram-II subtype was represented by low scores for stimulatory factors (IGS1, IGS3, IGS4, IGS5) and TNB (IGS2) and high scores for inhibitor molecules. The immunogram-III subtype presented high scores for the recognition of tumor cells and moderate scores for other factors (Fig. 1B). Favorable overall survival (OS) was observed for immunogram-I, and poor OS was observed for immunogram-II. Patients with immunogram-III and immunogram-IV patterns had OS values between those with immunogram-I and immunogram-II patterns (Fig. 1C). Furthermore,the result of LIRI-JP cohort was similar to TCGA cohort. The immunogram patternsof LIRI-JP HCC cohorts were separated into four clusters (termed immunogram-I to immunogram-IV, Additional file 2: Fig. S1A) by NMF clustering analysis. Favorable overall survival (OS) was observed in patients with immunogram-I patterns and poor OS was observed in patients with immunogram-II, immunogram-III and immunogram-IV patterns (Additional file 2: Fig. S1B).

These four immunogram subtypes comprised 32 cancer types in the TCGA dataset (Fig. 1D). Further analysis of the relationship between immunogram subtype and cancer type revealed that most cancer types were enriched in a given immunogram (Fig. 1E). The immunogram-I subtype was enriched in thymoma (THYM), lymphoid neoplasm diffuse large B-cell lymphoma (DLBC), kidney renal clear cell carcinoma (KIRC), testicular germ cell tumors (TGCTs), mesothelioma (MESO) and lung adenocarcinoma (LUAD). The proportion of immunogram-II tumors was high in uterine carcinosarcoma (UCS), ovarian serous cystadenocarcinoma (OV), uveal melanoma (UVM), sarcoma (SARC), and adrenocortical carcinoma (ACC). The immunogram-III subtype was enriched in UVM, rectum adenocarcinoma (READ), colon adenocarcinoma (COAD), cervical squamous cell carcinoma and endocervical adenocarcinoma (CESC). The immunogram-IV subtype was enriched in brain lower grade glioma (LGG), prostate adenocarcinoma (PRAD), uterine corpus endometrial carcinoma (UCEC), and glioblastoma multiforme (GBM). We also investigated the percentages of four immunogram subtypes among different clinical stages. The results indicated that the proportion of Immunogram-I decreased gradually, while the proportion of Immunogram-II, III, and IV showed an increasing trend in the progression from stage I to stage IV tumors (Fig. 1F).

### The immune features of the four immunogram subtypes

The tumor microenvironment is characterized by a number of innate and adaptive immune cell subpopulations, some of which show phenotypic plasticity and possess memory capabilities [15]. Therefore, we speculated that the composition of immune cells is distinct among the four immunogram patterns, which may contribute to the differences in phenotypes.

**Fig. 2:**
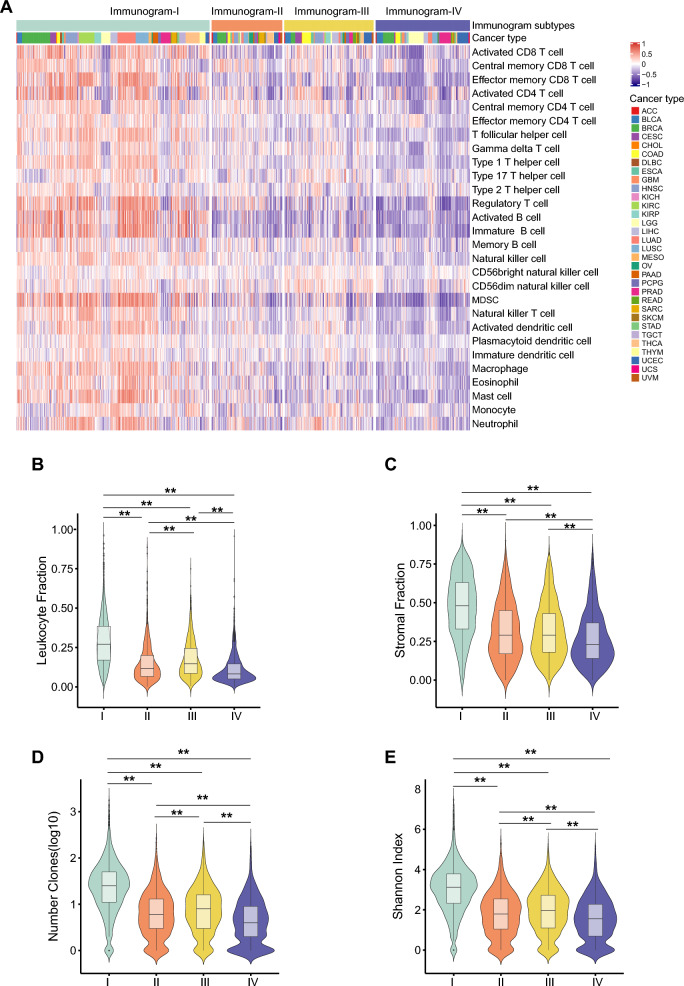
The immune features of four immunogram subtypes.** A** The relative abundance of 28 immune cell subsets that infiltrated the tumor was evaluated with the sample-level gene set enrichment method (GSVA) from the tumor RNA-Seq data. **B**–**E** The leukocyte fraction (**B**), stromal fraction (**C**), number of TCR clones (**D**), and Shannon index of TCRs (**E**) in the four immunogram subtypes (*P < 0.05, **P < 0.01)

To validate our hypothesis, the relative abundance of 28 immune cell subsets that infiltrated the tumor was evaluated in tumors with the four immunogram subtypes. Immunogram-I tumors showed a high level of infiltrating immune cells (Fig. 2A–C), high numbers of unique T cell receptor (TCR) clonotypes and a high Shannon index for TCRs (Fig. 2D, E). In contrast, immunograms II, III, and IV showed low levels of infiltrating immune cells, low numbers of unique TCR clonotypes and a high Shannon index for TCRs (Fig. 2D, E).

**Fig. 3:**
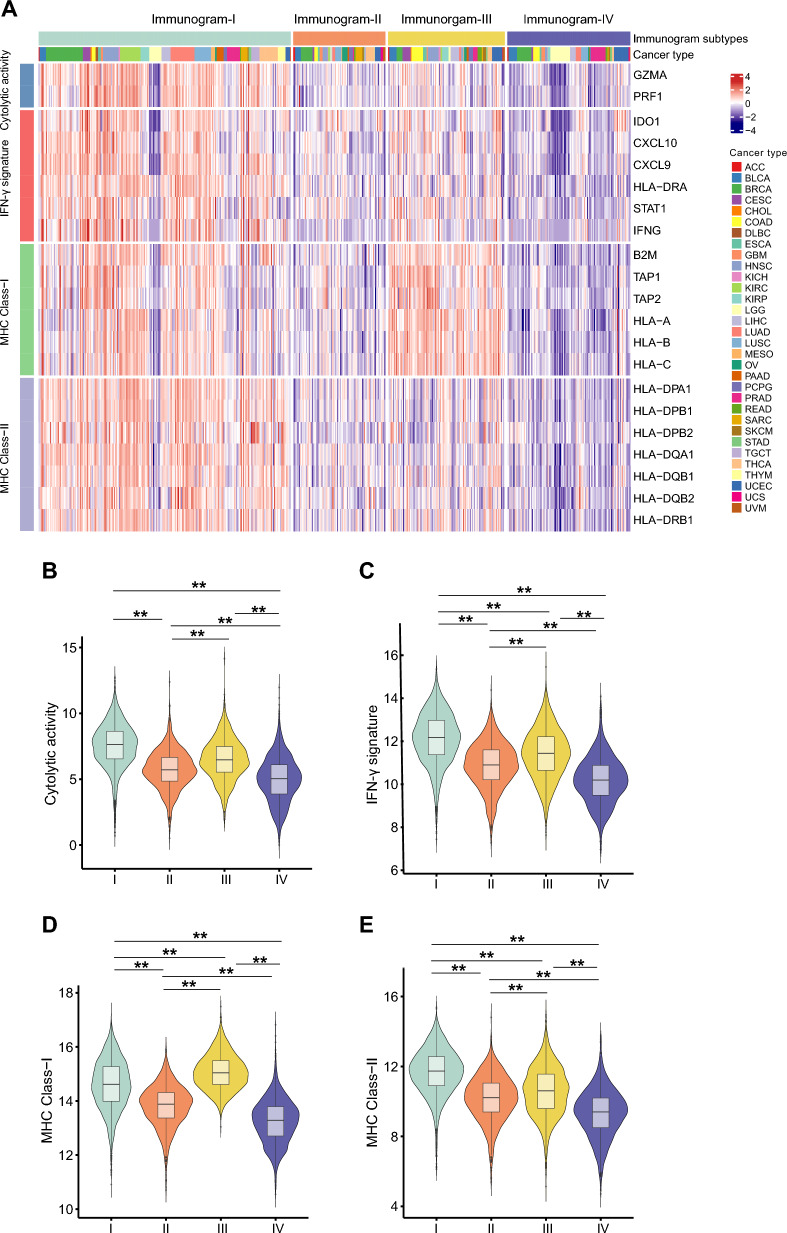
Expression of immune signatures in patients with the four immunogram patterns.** A** Heatmap of immune signatures including cytolytic activity, IFN-γ signature, MHC class-I and MHC class-II in tumors with the four immunogram patterns. **B**–**E** Violin plot of immune signatures including cytolytic activity (**B**), IFN-γ signature (**C**), MHC class-I (**D**) and MHC class-II (**E**) across the four immunogram subtypes (*P < 0.05, **P < 0.01)

Concurrently, the expression of immune signatures, including cytolytic activity, IFN-γ signature, major histocompatibility complex (MHC) class-I, and MHC class-II, immunoinhibitory and immunostimulatory molecules was increased in immunogram-I tumors (Fig. 3, Additional file 3: Fig S2). In particular, the upregulated expression of MHC class-I was observed in immunogram-III tumors (Fig. 3A, D). In addition, we observed higher levels of immune inhibitor molecules, including TGF-β1, TGF-β2, TGF-β3 and IL-10 in immunogram-I and immunogram-II tumors (Additional file 4: Fig S3).

Recent 10 years, the progress shed light on that the importance T cells within tumor microenvironment (TME), which included T cell migration into tumor through stroma, interaction with intratumoral immune cells, maintained effector state and function. Recently, Ira Mellman proposed that these steps of T cell in TME should be updated as the cancer-immunity subcycle [5]. Theoretically, TME phenotype may influence on the cancer-immunity cycle. Interestingly, we found that four immunogram subtypes which represented major visualization patterns of classical cancer-immunity cycle characterized by distinct TME phenotype. As shown in Additional file 5: Fig. S4), we found that high frequency (73.5%) of “desert” TME phenotype enriched in immunogram IV tumor. Oppositely, the lowest frequency “desert” phenotype enriched in immunogram I tumor. Meanwhile, relatively high frequency of “Immune Enriched non Fibrotic” enriched in immunogram I tumor. Immunogram II represented with high frequency of “Fibrotic” TME phenotype. Moreover, we found that immunogram I tumors were characterized by “IFN-γ dominant (C2)” and “inflammatory (C3)” phenotype. Immunogram II and Immunogram IV tumors represented high frequency of “wound healing (C1)” phenotype. Immunogram III represented high frequency of “IFN-γ dominant (C2)” phenotype (Additional file 5: Fig. S4B).

Collectively, these findings suggested that immunogram-I is characterized by ‘hot’ tumor features with activated and exhausted immune patterns. Immunogram-II, immunogram-III and immunogram-IV are characterized by ‘cold’ tumor features with an immunosuppressive phenotype.

### Distinct patterns of clonal selection in tumors with the four immunogram subtypes

**Fig. 4:**
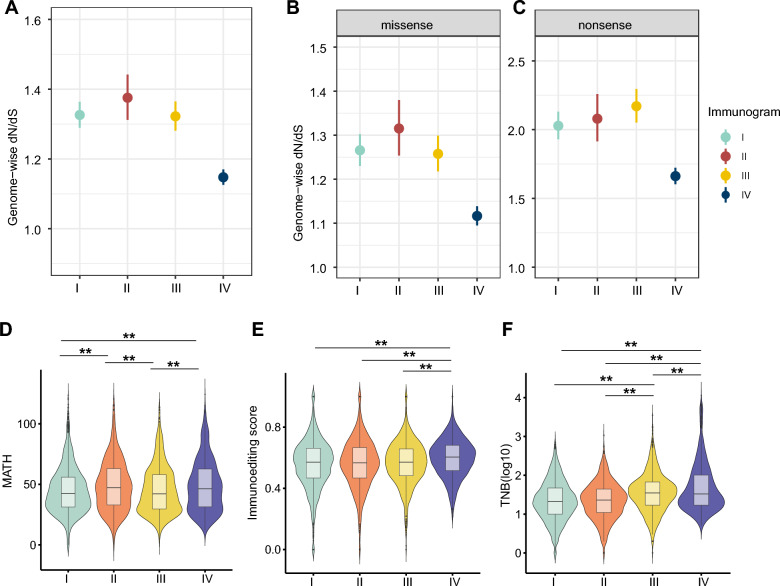
Distinct pattern of clonal selection for the four immunogram subtypes.** A**–**C** The dN/dS ratios for 715 known cancer-related genes in four immunogram subtypes considering all nonsynonymous (**A**), missense (**B**), and nonsense (**C**) mutations. **D**–**F** The values for MATH (**D**), immunoediting score (**E**), and TNB (**F**) in four immunogram subtypes (*P < 0.05, **P < 0.01)

A previous study showed that the immune system presents strong selection pressure during cancer evolution [17]. However, the impact of immunograms on clonal selection remains unclear. To evaluate the selection pressure of cancer evolution, we measured the dN/dS ratio (the ratio of nonsynonymous mutations to synonymous mutations) in 715 known cancer-related genes, which were derived from the Cancer Gene Census of the Cosmic database. Consistent with a previous study, dN/dS ratios were all greater than 1 in the four immunogram subtypes and presented as positive selection of the cancer immunogram. However, we found that the dN/dS ratio varied in tumors with the four immunogram subtypes (Fig. 4A–C). Immunogram-IV was characterized by a low dN/dS ratio for all nonsynonymous, missense, and nonsense mutations in 715 known cancer genes (Fig. 4A–C). In contrast, immunogram-II showed the highest dN/dS ratio for both nonsynonymous and missense tumor mutations in the four immunogram subtypes (Fig. 4A–C). Notably, Immunogram-I, with favorable OS, showed a moderate dN/dS ratio compared to other immunogram subtypes (Fig. 4A–C). To further validate our findings, we evaluated the dN/dS ratio in a re-sampling dataset for 715 genes (500 iterations), and found that the dN/dS ratios for 715 known cancer-related genes were higher than those for 715 randomly selected genes in all four immunogram subtypes (Additional file 6: Fig. S5). It suggested 715 known cancer-related genes under positive selection of cancer evolution.

Additionally, we found that immunogram-II and immunogram-IV both had high tumor heterogeneity reflected by high mutant-allele tumor heterogeneity (MATH) scores compared to other immunogram subtypes (Fig. 4D). However, clonal selection patterns were distinct between immunogram-II and immunogram-IV tumors. In particular, we found that although immunogram-IV tumors had low levels of positive selection, they were characterized by a high TNB. This was due to the low immune editing ability of immunogram-IV tumors (Fig. 4E). In contrast, immunogram-II tumors had high levels of positive selection and a low TNB (Fig. 4A–C, F).

### The molecular features of the four immunogram subtypes

**Fig. 5:**
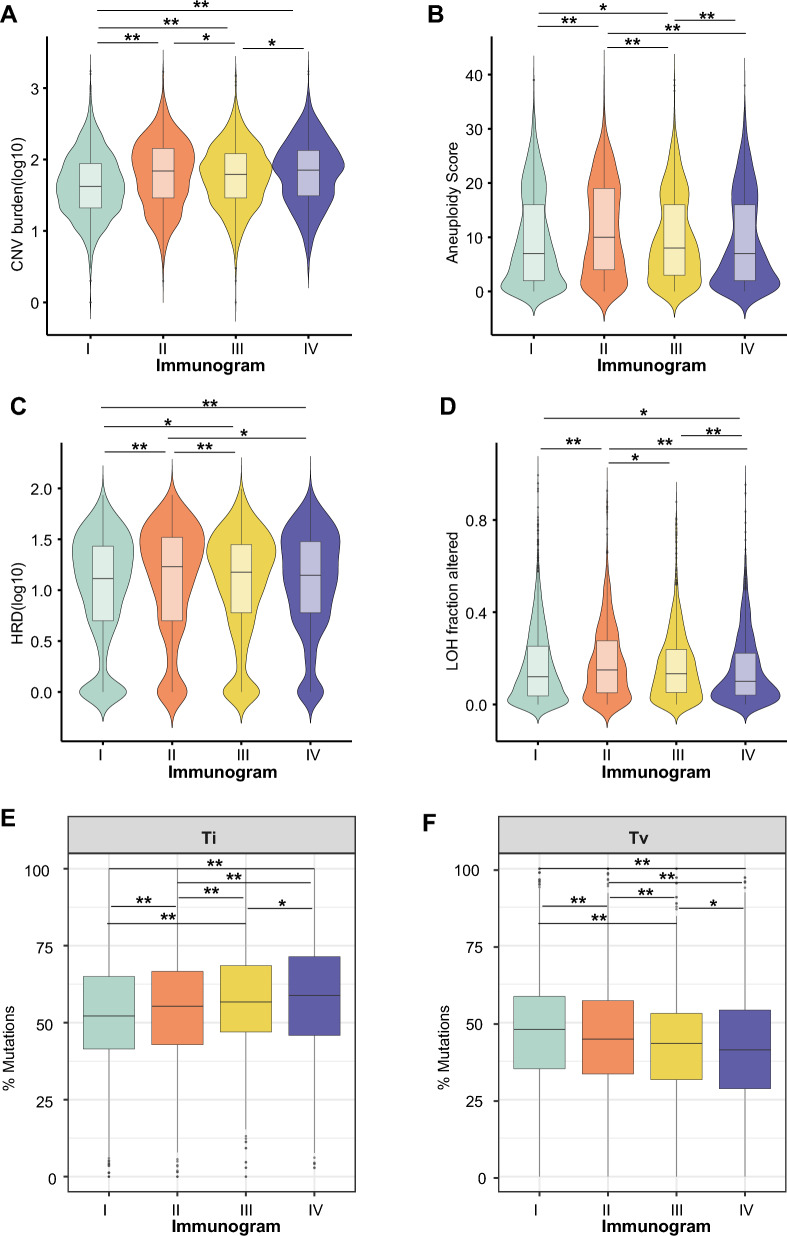
The molecular features of the four immunogram subtypes.** A**–**D** CNV burden (**A**), aneuploid score (**B**), HRD score (**C**) and LOH score (**D**) across the four immunogram subtypes. **E**, **F** The percentage of transition (Ti) mutations (**E**) and transversion (Tv) mutations (**F**) in the four immunogram subtypes (*P < 0.05, **P < 0.01)

Previous studies have shown a close relationship between the genotype and immunophenotype of tumors [7, 18]. However, the genomic features of tumors of different immunogram subtypes are unknown. Here, we examined genomic features, including mutation patterns, copy number variation (CNV) burden, aneuploid score, homologous recombination deficiency (HRD) score and loss of heterozygosity (LOH) score, across tumors of the four immunogram subtypes. The results showed that the genomic features varied among tumors with four immunogram subtypes. Immunogram-II was characterized by high CNV, aneuploid, HRD and LOH scores (Fig. 5A–D). In contrast, immunogram-I showed low levels of CNV, aneuploid, and HRD (Fig. 5A–C). Immunogram-IV showed low LOH scores (Fig. 5D). In addition, immunogram-IV exhibited a high percentage of transition (Ti) mutations and immunogram-I presented a high percentage of transversion (Tv) mutations (Fig. 5E, F).

### The ability of immunogram patterns to predict the response to ICI therapy

**Fig. 6:**
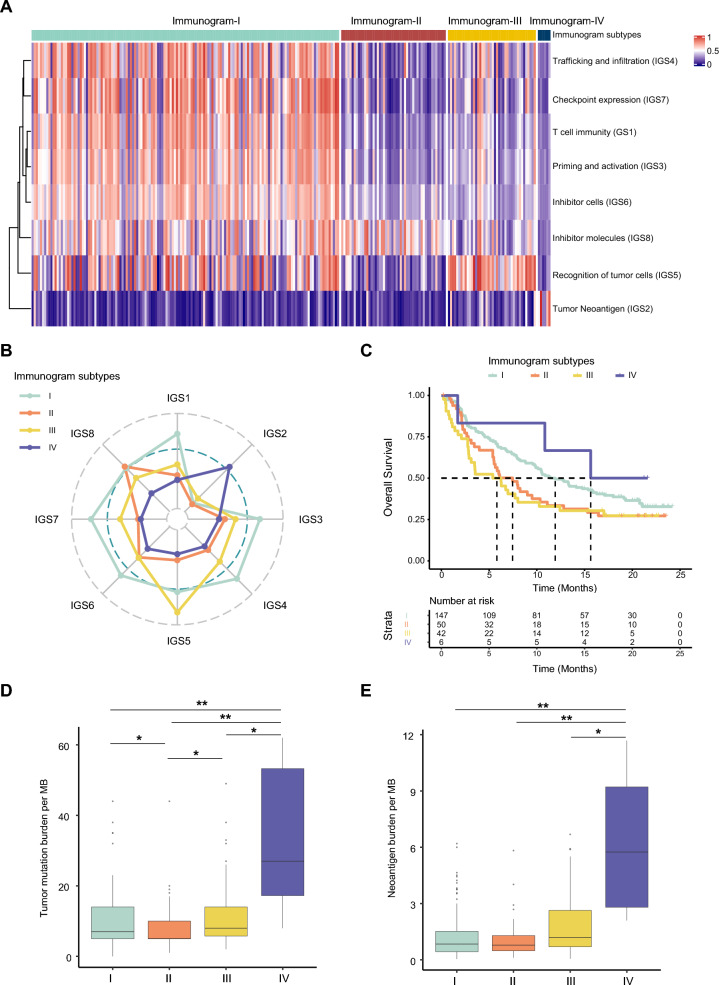
The efficacy of immunogram patterns predicts the response to ICI therapy.** A** NMF clustering analysis of immunograms based on the eight axes of the IGS in the IMvigor210 cohort. **B** Radar plot of the four immunogram patterns in the Imvigor210 cohort. **C** Kaplan–Meier curves for OS of patients in the Imvigor210 cohort stratified by the four immunogram subtypes. **D**, **E** The TMB (**D**) and TNB € of patients with the four immunogram patterns in the Imvigor210 cohort (*P < 0.05, **P < 0.01)

In view of the cancer-immunity cycle, patients with distinct cancer immunogram patterns may have different responses to immune therapy. We extended our investigation to the association between immunogram patterns and the efficacy of ICIs in the metastatic urothelial cancer (mUC) cohort (IMvigor210 cohort) [9, 19]. In this mUC cohort, the four cancer immunogram patterns were illustrated by heatmaps and radar plots (Fig. 6A, B). Interestingly, favorable OS was observed in mUC patients with immunogram-I (n = 147, median OS: 11.93 months) and immunogram-IV (n = 6, median OS: 15.64 months) subtypes after ICI therapy. In contrast, poor OS was observed in mUC patients with immunogram-II (n = 50, median OS: 7.46 months) and immunogram-III (n = 42, median OS: 5.82 months) subtypes after ICI therapy (Fig. 6C).

mUC patients with immunogram-I tumors presented high scores for both stimulatory and inhibitory factors but with low TMB and TNB scores (Fig. 6A, B, D and E). Conversely, mUC patients with immunogram-IV tumors had low scores for both stimulatory and inhibitory factors but high TMB and TNB scores (Fig. 6A, B, D and E). mUC patients with immunogram-II tumors showed high scores for IGS8 (inhibitor molecules) and low scores for stimulatory factors, TMB and TNB. mUC patients with immunogram-III tumors showed high IGS5 (recognition of tumor cells) scores and low stimulatory factor, TMB and TNB scores (Fig. 6A, B, D and E).

Furthermore, we analyzed the factors including clinical character, tumor features, immune features that may affect the OS after ICI therapy in IMvigor210 cohort. Firstly, univariate Cox regression analysis indicated that baseline ECOG score, metastatic disease status, immunogram subtype, PD-L1 Expression (IC levels), immune phenotype, immune checkpoint expression, MHC class I antigen presenting machinery expression (APM), WNT signaling level, Lund molecular subtype, TNB levels and TMB levels were significantly associated with OS of ICI therapy (Additional file 7: Fig. S6A). Secondly, the multivariate Cox regression analysis indicated that baseline ECOG score (ECOG score = 1, ECOG score = 2), immunogram subtype (Immunogram III), Lund molecular subtype (basal/SCC-like, SCCL) were risk factor for OS (HR > 1, P < 0.05). PD-L1 Expression (IC levels2+) and APM were protective factors for OS (HR < 1, P < 0.05) (Additional file 7: Fig. S6B). Moreover, we compared the tumor molecular features among the immunogram subtypes. We found that basal/SCC-like (SCCL) subtypes that risk factors for OS were enriched in Immunogram III tumors Additional file 8: Fig. S7A. Compared with other immunogram subtypes, WNT signaling score was highest in immunogram II tumors (Additional file 8: Fig. S7B).

In addition, we further investigate the association between immunogram subtype and the response to PD-1 inhibition (pembrolizumab) in metastatic gastric cancer (mGC) (ClinicalTrails.gov, NCT#02589496) [10]. As shown in Additional file 9: Fig.S8, we found that all mGC patients with immunogram I phenotype had a high response rate to PD-1 inhibition (fraction of patients: CR, 50%; PR, 50%). Conversely, mGC patients with immunogram II, immunogram III and immunogram IV subtypes showed lower response rate to PD-1 inhibition (Additional file 9: Fig. S8).

## Discussion

**Fig. 7:**
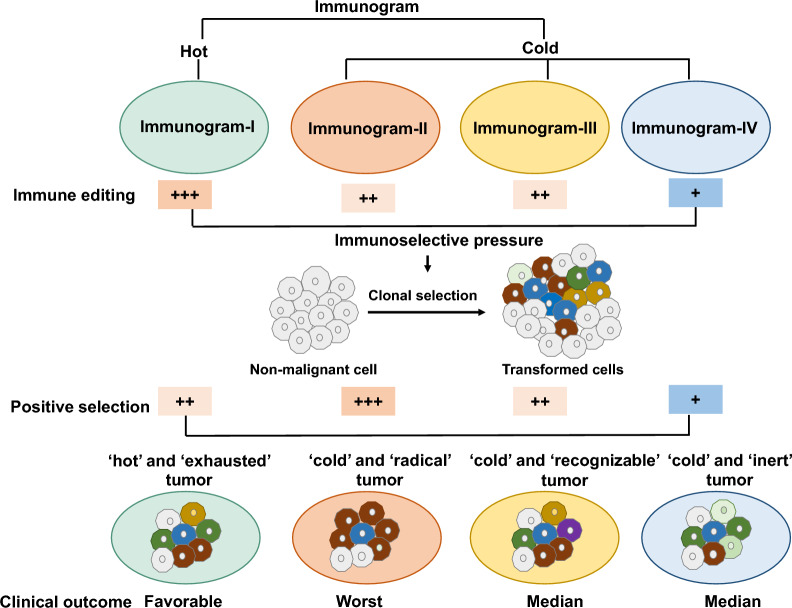
Immunogram defines four cancer-immunity cycle phenotypes with distinct clonal selection patterns across solid tumors. An overview of key findings from this study

A comprehensive understanding of cancer-immunity interactions is vital for developing novel antitumor drugs and implementing clinical strategies. Based on the cancer-immune cycle theory, this study illustrated immunogram patterns in 8460 cancer patients to visualize the state of cancer-immune system interactions (Fig. 7). We further analyzed the interplay between the cancer clonal selection pattern and cancer immunograms. Moreover, the efficacy of ICI therapy in different cancer immunogram patterns was evaluated in our study (Fig. 6, Additional file 7: Fig. S7).

Recently, the update of the cancer-immune cycle theory emphasized the iterative nature of the antitumor immune response adapting to tumor evolution [5]. The weakness in any step of cancer-immune cycle will become the rate-limiting lending the tumor escaping from immune system. The present study illustrated major four cancer-immunity cycle patterns across solid tumors, which uncovered solid tumors’ major shortness in cancer-immune cycle. These findings provided resources to implement therapeutic strategies for each immunogram subtype. Immunogram-I patterns were characterized by ‘hot’ and ‘exhausted’ features, which showed both high levels of immune infiltration and checkpoint inhibitor expression. ICI therapy could reinvigorate and potentially enhance the pre-existing anticancer immune response. We found that mUC patients with immunogram-I had a longer median OS than patients with immunogram-II and immunogram-III. mGC patient with immunogram-I also had a higher response rate to PD-1 inhibition (pembrolizumab). In particularly, immunograms II, III and IV exhibited distinct immunosuppressive patterns of ‘cold’ tumor features. Immunogram-II patterns were characterized by ‘cold’ and ‘radical’ features, which represent a high rate of cancer evolution and may result in the accumulation of more deleterious mutations. Meanwhile, the immune patterns of Immunogram-II showed low levels of immune activation factors but high levels of inhibitor molecules, including TGF-β and IL-10. Additional, immunogram II characterized by high frequency of “Fibrotic” TME phenotype. These factors leaded to the outcome that immunogram II tumors had a high ability to escape immune attack. These immunosuppressive features also may be the reason that the mUC and mGC patients with immunogram II phenotype have worse response after PD-1/PD-L1 inhibition monotherapy. Therefore, the combination strategy of both activating the antitumor immune response and neutralizing immune inhibitors may be effective. Immunogram-III patterns were characterized by ‘cold’ and ‘recognizable’ features, which showed low levels of other dimensions of the antitumor response but upregulated expression of only MHC-I class expression, suggesting that the immune systems of the immunogram-III subtype have the potential to recognize tumor neoantigens. Accordingly, the strategy for the immunogram-III subtype should focus on improving other weak points of the immunogram, including T cell immunity, priming and activation, trafficking and infiltration. Immunogram-IV patterns are characterized by ‘cold’ and ‘inert’ features, which represent overall immunosuppression and a low rate of cancer evolution and accumulate more tumor neoantigens that are more visible to the immune system. Our results showed that mUC patients with immunogram IV disease had the longest OS with ICI therapy among the four major immunogram subtypes.

Admittedly, except for immune features, tumor features also contributed to the clinical outcome of ICI therapy. Immunogram I and immunogram IV tumors have distinct immune features, but they have similar OS after ICI therapy in mUC patients. That may be explained by the distinct Lund molecular type between Immunogram I and immunogram IV tumors. Although the Immunogram I tumors represent high levels of immune infiltration and checkpoint inhibitor expression. However, compared with immunogram IV, immunogram I tumors also had a higher percentage of SCCL subtypes which was associated with poor OS in mUC patients after ICI therapy. For immunogram II and immunogram III tumors, tumor features and immunosuppressive features may both contribute to the poor OS after PD-1/PD-L1 inhibition monotherapy. Immunogram II tumors represented high levels of WNT signaling score and CNV scores. Immunogram III tumors had a high percentage of SCCL subtypes in mUC patients. These tumor features were associated with resistance to ICI therapy [20, 21].

Cancer is an end product of somatic evolution [14]. Cancer clones have advantages in supporting cell survival after positive selection [14]. And the level of positive selection reflected the rate of cancer evolution. Our findings indicated that the different immunograms may exert distinct levels of pressure on clonal selection. In addition to representing different levels of immunoselective pressure, the four immunogram subtypes represented distinct clonal selection patterns (Fig. 7). A previous study showed that tumor heterogeneity fosters cancer evolution [22]. Interestingly, we found that although both immunogram-II and immunogram-IV were characterized by high levels of tumor heterogeneity, the two subtypes showed different patterns of clonal selection. This difference may be because immunogram-II tumors have higher levels of tumor immune cell infiltration and immune editing ability. In response to high levels of immune editing, tumors present high levels of positive clonal selection to accumulate advantageous tumor clones that could escape immune editing. In addition, we found that the TNBs were different within the four subtypes, which may be associated with the distinct patterns of interplay between immune editing and cancer evolution among immunogram subtypes. A previous study indicated that tumor cell clones with low immunogenicity have advantages in escaping from immune attack and are therefore selected, while highly immunogenic tumor clones are eradicated [1]. Highly immunogenic tumor clones are eradicated during immunoediting, and high levels of positive selection may lead to the selection of tumor clones with low immunogenicity that are invisible to the immune system and have a survival advantage. Therefore, it may be speculated that immunogram-II showed a high level of positive selection and immune editing that may result in fewer tumor clones with high neoantigen loads. In contrast, immunogram-IV was characterized by a low level of positive selection, and immune editing contributed more tumor clones with high neoantigen loads. From a evolution point of view, the interaction between tumor and immunity characterized by “generation and restriction”, which is the foundation of the coevolution between them. Our findings were just snapshots of constant motion of antitumor immune response and tumor evolutions. The further research should be designed to uncover the dynamic interaction between the cancer and immunity.

In summary, our study further illustrated the four major patterns of cancer-immunity cycle among pancancer. For each immunogram subtype, an effective strategy should be implemented to improve the weak point of the immunogram and then strengthen the antitumor immune response. Our findings may contribute to optimizing anticancer strategies.

### Supplementary Information

**Additional file 1: Table S1.** Summary of immunogram subtype for each analyzed tumor in TCGA cohort. Related to Fig. 1. **Table S2.** Summary of clinical information for each analyzed tumor in TCGA cohort. Related to Fig. 1. **Table S3.** Summary of clinical information and immunogram subtype for each analyzed tumor in IMvigor210 cohort. Related to Fig. 6. **Table S4.** Summary of clinical information and immunogram subtype for each analyzed tumor in metastatic gastric cancer cohort. Related to Figure S7. **Table S5.** Summary of clinical information and immunogram subtype for each analyzed tumor in LIRI-JP cohort. Related to Additional file 2: Fig S1 Table S5 Summary of clinical information andimmunogram subtype for each analyzed tumor in metastatic gastric cancer cohort. Related to Additional file 9: Fig. S8.

**Additional file 2: Figure S1.** Immunogram subtypes and prognosis in LIRI-JP HCCcohortfrom ICGC database. **A**, The radar plot showed that the mmunogram patterns of the four clusters were distinct. The axes of the radar chart were generated according to the median IGS for the four immunogram subtypes. **B** Kaplan-Meier curves for the OS of HCC patients in the LIRI-JP cohort stratified by the four immunogram subtypes. The log-rank test yielded *P* = 0.036.

**Additional file 3: Figure S2.** Expression of MHC and immunomodulatory molecules in patients with the four immunogram patterns. Heatmap of the expression of MHC class-I, MHCclass-II, immunoinhibitory and immunostimulatory molecules in tumors with the four immunogram patterns.

**Additional file 4: Figure S3.** Expression of immune inhibitor molecules in patients with the four immunogram patterns. **A**–**D** Violin plot of immune inhibitor molecules including TGF-β1 (**A**), TGF-β2 (**B**), TGF-β3 (**C**) and IL-10 (**D**) across the four immunogram subtypes (*P < 0.05, **P < 0.01).

**Additional file 5: Figure S4.** The percentage of classical tumor immunogical type in four immunoram subtype of solid tumors. **A** The percentage of tumor microenvironment (phenotypes in four immunoram subtype of solid tumors. *B* The percentage of immune subtypes in four immunoram subtype of solid tumors. Immune subtypes C1, Wound healing C2, IFN-γ dominant; C3, Inflammatory; C4, Lymphocyte depleted C5, Immunologically quiet C6, TGF-β dominant.

**Additional file 6: Figure S5**. The distributions of dN/dS ratios in the four immunogram subtypes. **A**-**C** The distributions of dN/dS ratios for the 715 randomly selected genes and 715 known cancer related genes in the four immunogram subtypes considering all nonsy nonymous (**A**), missense (**B**), and nonsense (**C**).

**Additional file 7: Figure S6.** Effect of clinical character, tumor features, and immune features on OS after ICI therapy in IMvigor210 cohort. **A** Univariate Cox regression analysis of factors effecting OS after ICI therapy in IMvigor210 cohort. **B** Multivariate Cox regression analysis of factors effecting OS after ICI therapy in IMvigor210 cohort For est plot for OS in subgroups. HR, hazard ratio CI, confidence intervals.

**Additional file 8: Figure S7.** The tumor features of mUC patients with four immunogram subtypes in IMvigor210 cohort. **A**, The fraction of patients of mUC patients with Lund molecular subtype among four immunogram subtypes. Lund molecular subtype: GU, genomically unstable; Inf, infiltrated; SCCL, basal/SCC-like; UroA, urothelial-like A; UroB, urothelial-like B. **B**, WNT signaling score in mUC tumors with four immunogram subtypes.

**Additional file 9: Figure S8.** The fraction of metastatic gastric cancer (mGC) patients response to PD1 inhibition (pembrolizumab) in four immunogram subtype. CR, complete response; PR, partial response; SD, stable disease; progressive disease.

## Data Availability

Data are available in a public, open access repository. The clinical data, RNA sequencing and WES data of the TCGA, LIRI JP cohort, IMvigor210, and metastatic gastric cancer cohort were available from a previously published study (TCGA: https://gdc.cancer.gov/about-data/publications/PanCan-CellOfOrigin, LIRI JP cohort https://dcc.icgc.org/, IMvigor210 cohort: http://research-pub.gene.com/IMvigor210CoreBiologies, metastatic gastric cancer cohort: European Nucleotide Archive, available under accession PRJEB25780).

## Abbreviations

ICI: Immune checkpoint inhibitor
IGS: Immunogram score
OS: Overall survival
NMF: Nonnegative matrix factorization
MHC: Major histocompatibility complex
HRD: Homologous recombination deficiency
CNV: Copy number variation
LOH: Loss of heterozygosity
TMB: Tumor mutation burden
TNB: Tumor neoantigen burden
mUC: Metastatic urothelial cancer
