# Supplementary material for: Immunogram defines four cancer-immunity cycle phenotypes with distinct clonal selection patterns across solid tumors
Source: J Transl Med. 2024 Jan 20;22:69. doi: 10.1186/s12967-023-04765-5 (PMC10799518; doi:10.1186/s12967-023-04765-5)
Supplement: Supplementary file 2 — Additional file 2: Figure S1. Immunogram subtypes and prognosis in LIRI-JP HCCcohortfrom ICGC database. A, The radar plot showed that the mmunogram patterns of the four clusters were distinct. The axes of the radar chart were generated according to the median IGS for the four immunogram subtypes. B Kaplan-Meier curves for the OS of HCC patients in the LIRI-JP cohort stratified by the four immunogram subtypes. The log-rank test yielded P = 0.036. [file 12967_2023_4765_MOESM2_ESM.pdf]

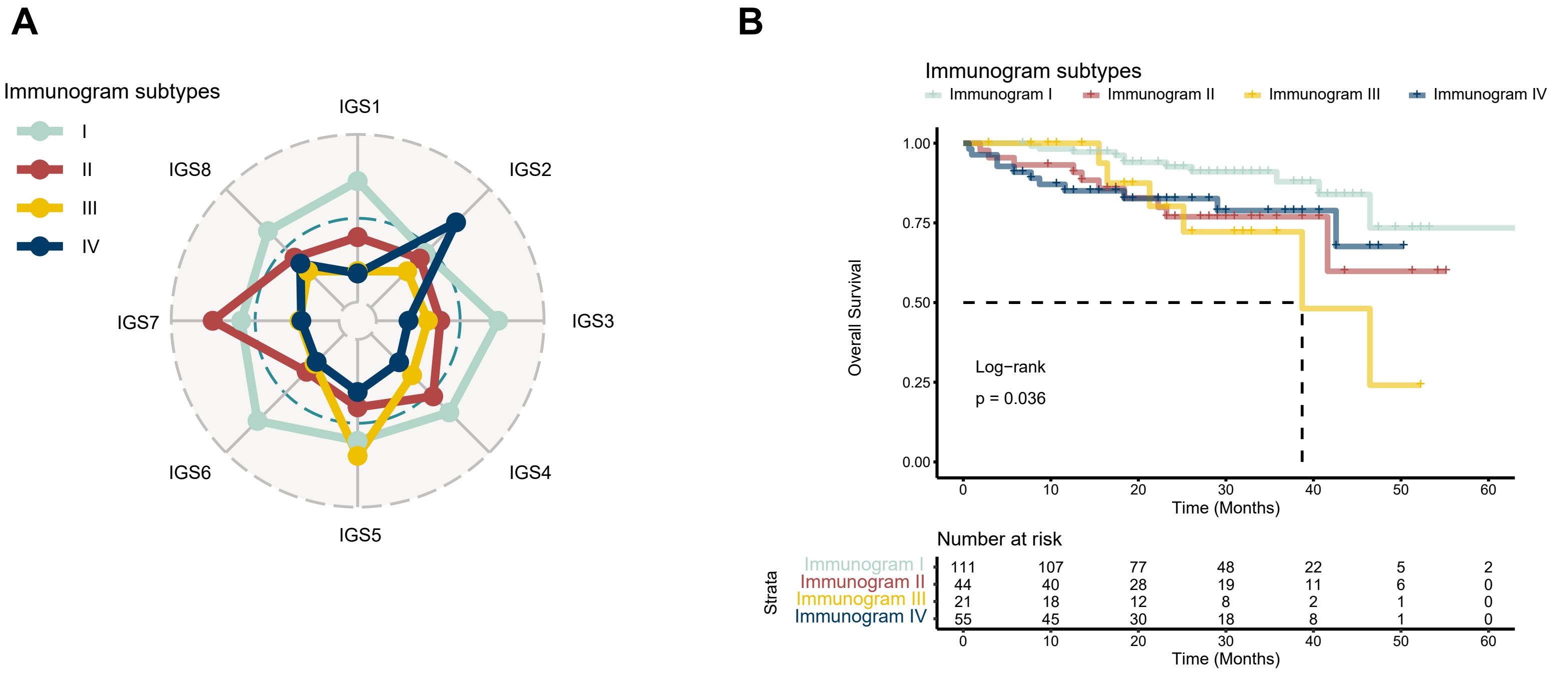

Supplementary Figure 1
