# Supplementary figures and images for: Immunogram defines four cancer-immunity cycle phenotypes with distinct clonal selection patterns across solid tumors
Source: J Transl Med. 2024 Jan 20;22:69. doi: 10.1186/s12967-023-04765-5 (PMC10799518; doi:10.1186/s12967-023-04765-5)

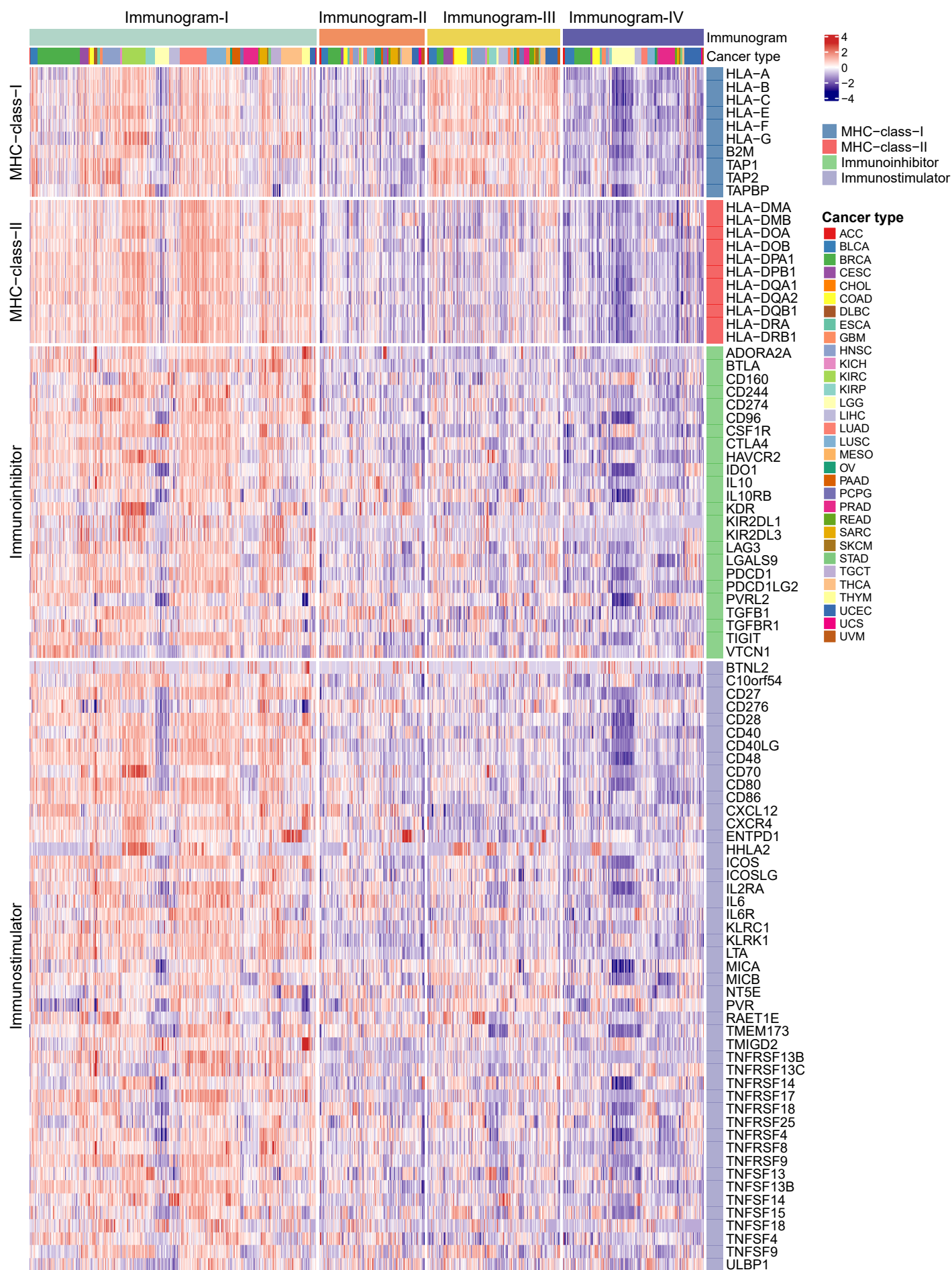

Supplementary Figure 2

Supplement: Supplementary file 3 — Additional file 3: Figure S2. Expression of MHC and immunomodulatory molecules in patients with the four immunogram patterns. Heatmap of the expression of MHC class-I, MHCclass-II, immunoinhibitory and immunostimulatory molecules in tumors with the four immunogram patterns. [file 12967_2023_4765_MOESM3_ESM.pdf]

**A**

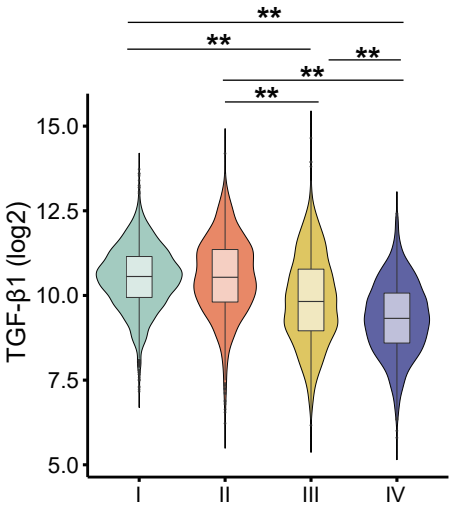

**B**

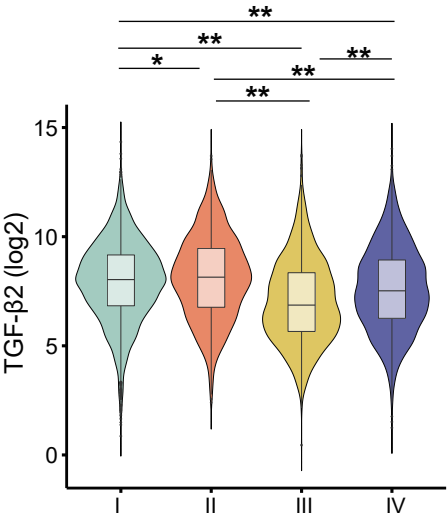

**C**

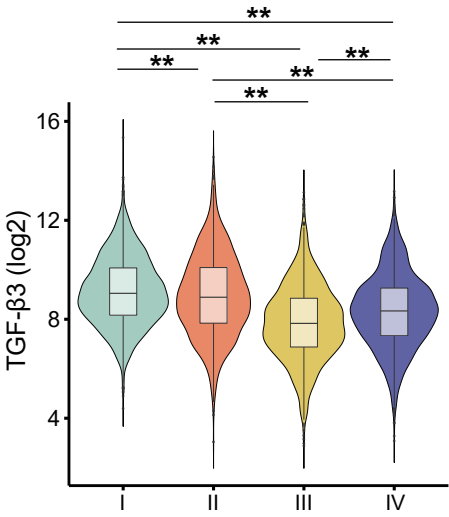

**D**

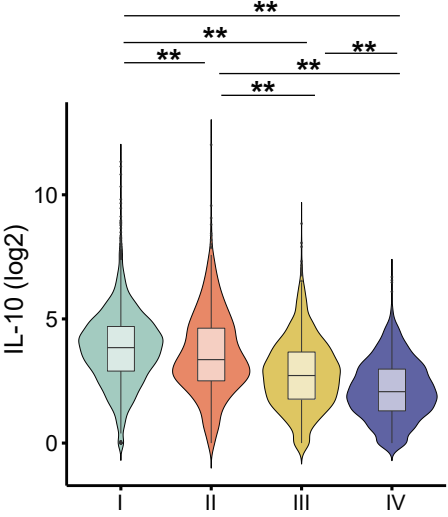

Supplement: Supplementary file 4 — Additional file 4: Figure S3. Expression of immune inhibitor molecules in patients with the four immunogram patterns. A–D Violin plot of immune inhibitor molecules including TGF-β1 (A), TGF-β2 (B), TGF-β3 (C) and IL-10 (D) across the four immunogram subtypes (*P < 0.05, **P < 0.01). [file 12967_2023_4765_MOESM4_ESM.pdf]

**A**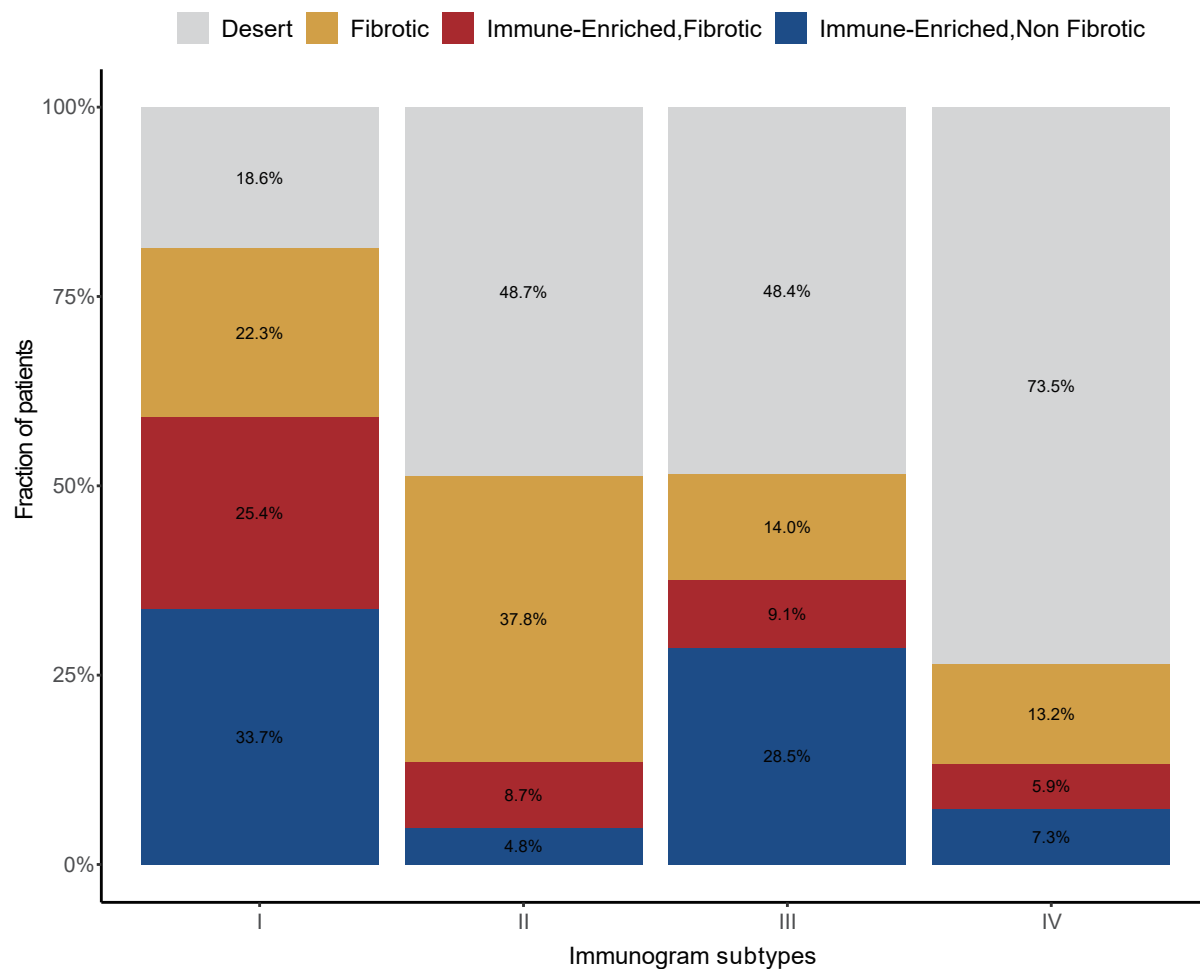**B**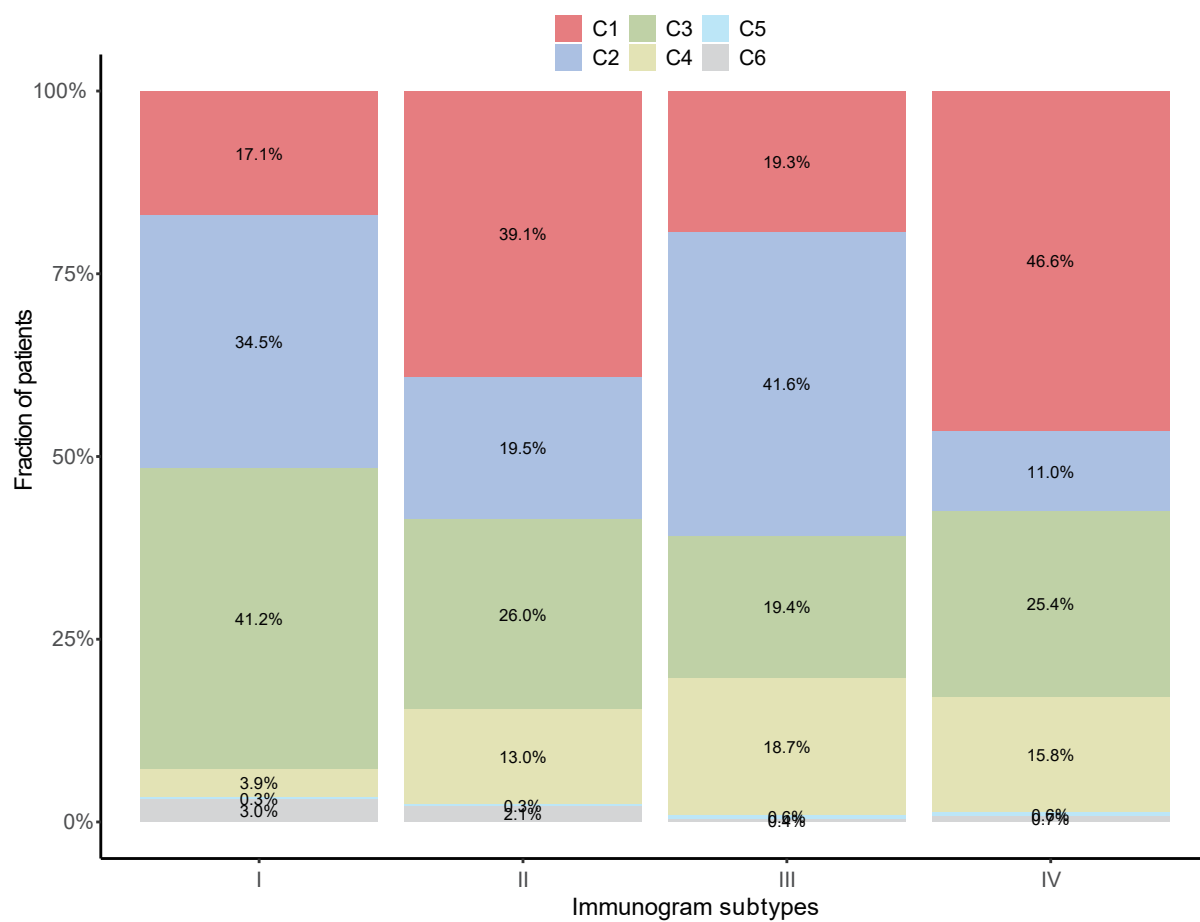**Supplementary Figure 4**

Supplement: Supplementary file 5 — Additional file 5: Figure S4. The percentage of classical tumor immunogical type in four immunoram subtype of solid tumors. A The percentage of tumor microenvironment (phenotypes in four immunoram subtype of solid tumors. B The percentage of immune subtypes in four immunoram subtype of solid tumors. Immune subtypes C1, Wound healing C2, IFN-γ dominant; C3, Inflammatory; C4, Lymphocyte depleted C5, Immunologically quiet C6, TGF-β dominant. [file 12967_2023_4765_MOESM5_ESM.pdf]

**A**

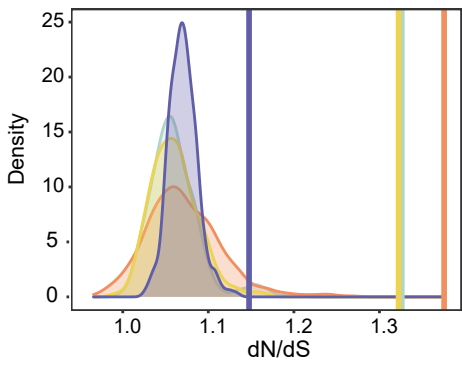

**B**

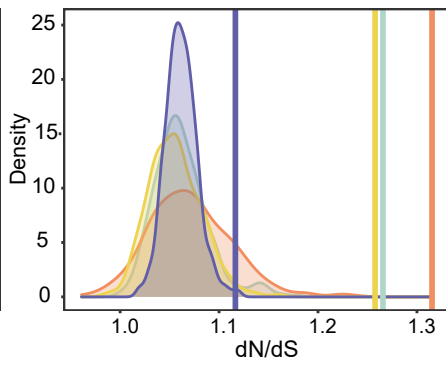

**C**

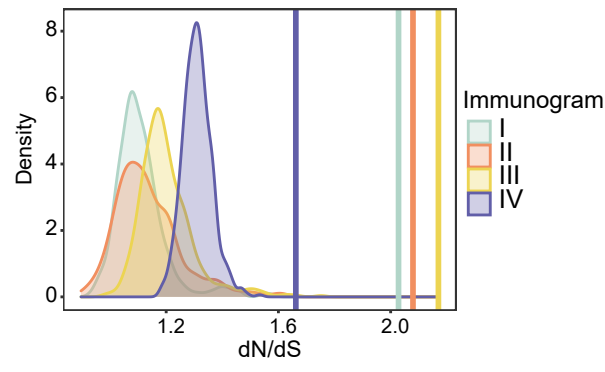

Supplement: Supplementary file 6 — Additional file 6: Figure S5. The distributions of dN/dS ratios in the four immunogram subtypes. A-C The distributions of dN/dS ratios for the 715 randomly selected genes and 715 known cancer related genes in the four immunogram subtypes considering all nonsy nonymous (A), missense (B), and nonsense (C). [file 12967_2023_4765_MOESM6_ESM.pdf]

**A**

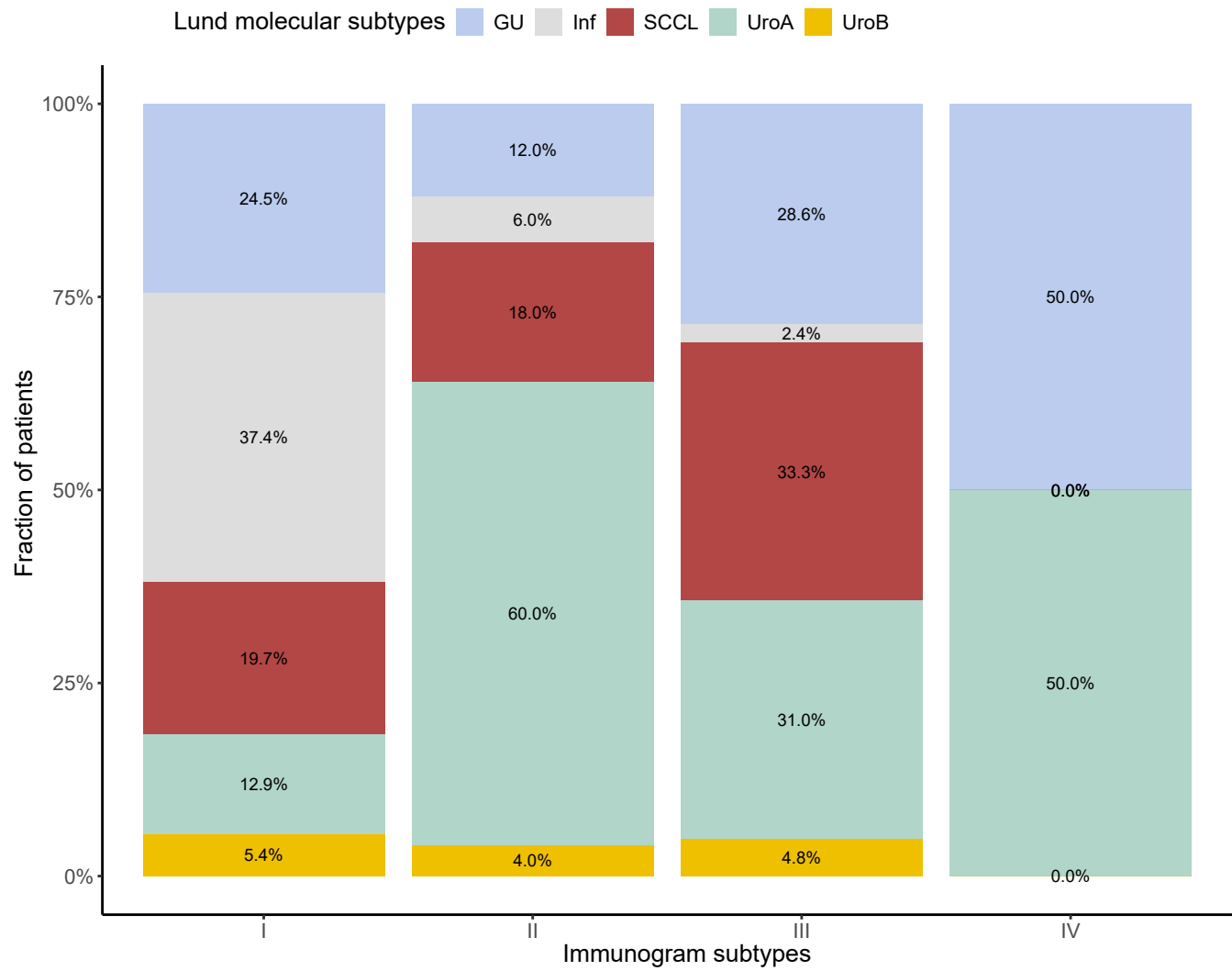

**B**

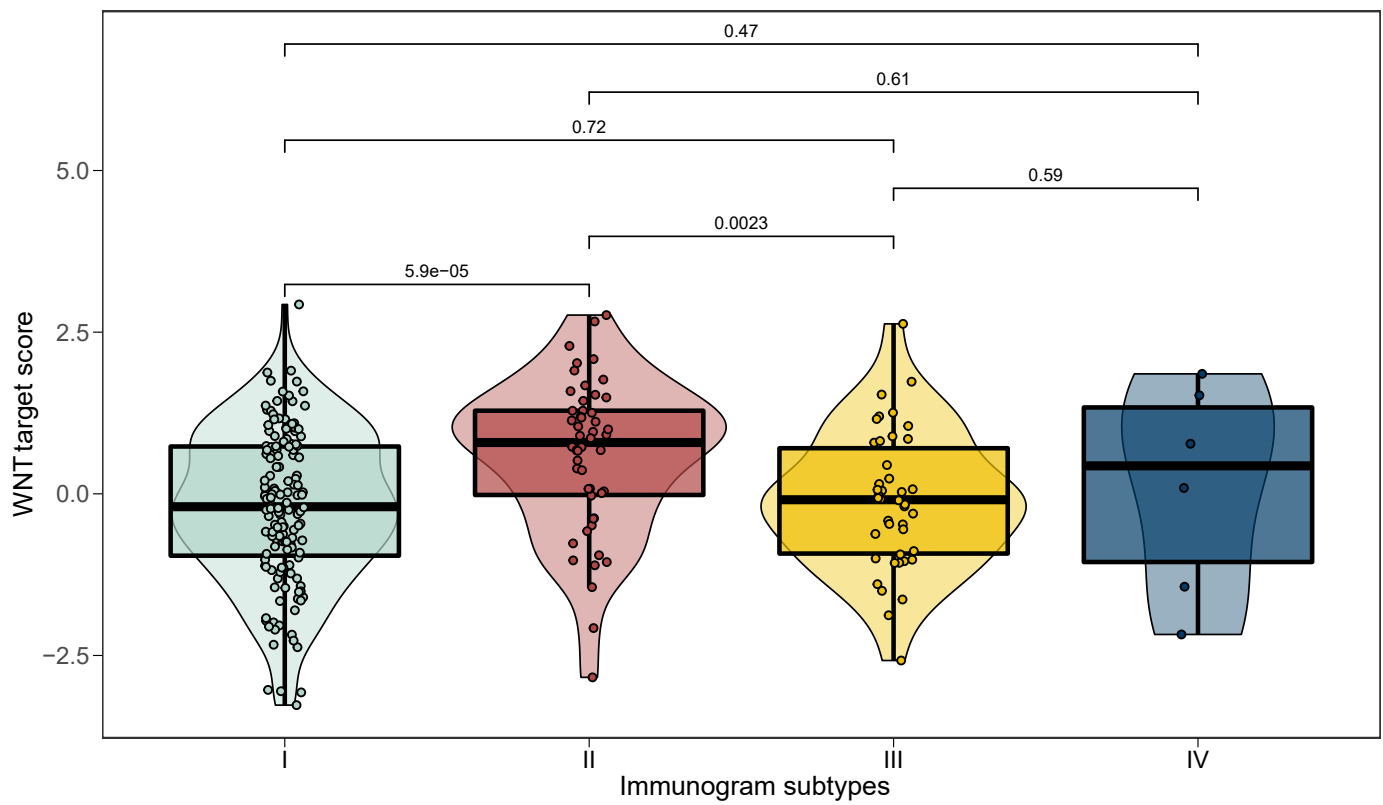

Supplement: Supplementary file 8 — Additional file 8: Figure S7. The tumor features of mUC patients with four immunogram subtypes in IMvigor210 cohort. A, The fraction of patients of mUC patients with Lund molecular subtype among four immunogram subtypes. Lund molecular subtype: GU, genomically unstable; Inf, infiltrated; SCCL, basal/SCC-like; UroA, urothelial-like A; UroB, urothelial-like B. B, WNT signaling score in mUC tumors with four immunogram subtypes. [file 12967_2023_4765_MOESM8_ESM.pdf]

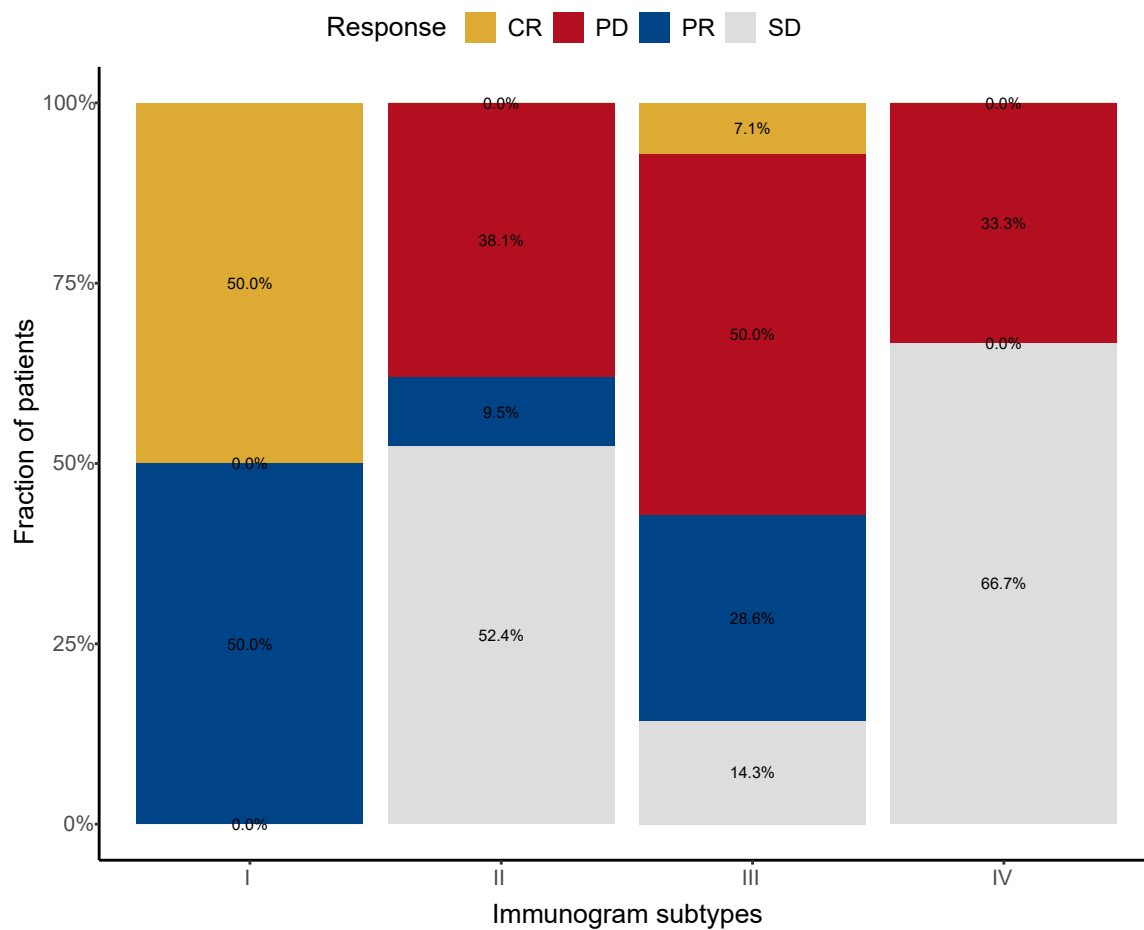

Supplement: Supplementary file 9 — Additional file 9: Figure S8. The fraction of metastatic gastric cancer (mGC) patients response to PD1 inhibition (pembrolizumab) in four immunogram subtype. CR, complete response; PR, partial response; SD, stable disease; progressive disease. [file 12967_2023_4765_MOESM9_ESM.pdf]
