# Supplementary material for: Immunogram defines four cancer-immunity cycle phenotypes with distinct clonal selection patterns across solid tumors
Source: J Transl Med. 2024 Jan 20;22:69. doi: 10.1186/s12967-023-04765-5 (PMC10799518; doi:10.1186/s12967-023-04765-5)
Supplement: Supplementary file 7 — Additional file 7: Figure S6. Effect of clinical character, tumor features, and immune features on OS after ICI therapy in IMvigor210 cohort. A Univariate Cox regression analysis of factors effecting OS after ICI therapy in IMvigor210 cohort. B Multivariate Cox regression analysis of factors effecting OS after ICI therapy in IMvigor210 cohort For est plot for OS in subgroups. HR, hazard ratio CI, confidence intervals. [file 12967_2023_4765_MOESM7_ESM.pdf]

A

Variable	p value	HR (95% CI)
Baseline ECOG score,1	<0.001	2.100(1.450−3.030)
Baseline ECOG score,2	0.013	2.400(1.210−4.780)
Metastatic status,LN Only	<0.001	0.310(0.170−0.570)
Metastatic status,Visceral	0.023	0.650(0.450−0.940)
Immunogram II	0.045	1.520(1.010−2.290)
Immunogram III	0.016	1.730(1.110−2.710)
IC Level, IC2+	0.001	0.470(0.300−0.730)
Immune phenotype, inflamed	0.046	0.610(0.380−0.990)
APM	0.024	0.910(0.840−0.990)
Immune checkpoint	0.004	0.900(0.840−0.970)
WNT target score	0.019	1.190(1.030−1.380)
Lund,SCCL	0.002	2.210(1.330−3.690)
Lund,UroA	0.016	1.830(1.120−3.000)
TMB level,medium	0.044	0.610(0.380−0.990)
TMB level,high	0.005	0.540(0.350−0.820)
TMB level,extremely high	<0.001	0.330(0.200−0.550)
TNB level,extremely high	<0.001	0.320(0.190−0.540)

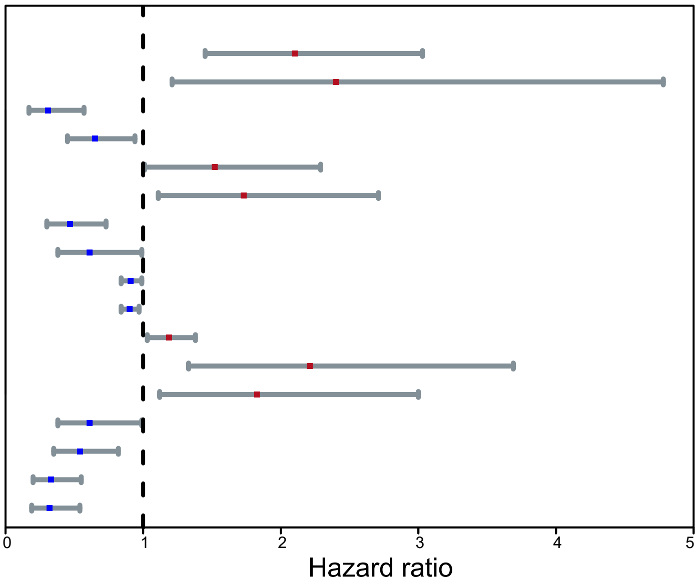

B

Variable		N	HR (95% CI)	p value
Baseline ECOG score	0	65	Reference	
	1	92	2.36 (1.51, 3.70)	<0.001
	2	14	4.18 (1.75, 10.00)	0.001
Metastatic status	Liver	57	Reference	
	LN Only	32	0.57 (0.29, 1.11)	0.098
	Visceral	82	0.73 (0.47, 1.11)	0.139
Immunogram subtypes	I	101	Reference	
	II	34	1.05 (0.58, 1.90)	0.873
	III	32	2.26 (1.08, 4.74)	0.031
	IV	4	1.31 (0.27, 6.35)	0.740
IC Level	IC0	36	Reference	
	IC1	66	0.85 (0.48, 1.53)	0.598
	IC2+	69	0.43 (0.21, 0.89)	0.022
Immune phenotype	desert	41	Reference	
	excluded	80	0.76 (0.43, 1.35)	0.351
	inflamed	50	0.86 (0.37, 1.97)	0.713
APM		171	0.80 (0.65, 0.99)	0.039
Immune Checkpoint		171	1.18 (0.97, 1.44)	0.099
WNT target score		171	1.09 (0.87, 1.37)	0.431
Lund	GU	43	Reference	
	Inf	38	0.94 (0.43, 2.05)	0.879
	SCCL	37	2.60 (1.30, 5.17)	0.007
	UroA	46	1.13 (0.56, 2.25)	0.735
	UroB	7	2.61 (0.84, 8.07)	0.096
TMB level	low	59	Reference	
	medium	26	0.92 (0.51, 1.66)	0.770
	high	51	0.64 (0.37, 1.09)	0.102
	extremely_high	35	0.73 (0.30, 1.79)	0.490
TNB level	low	44	Reference	
	medium	40	1.35 (0.78, 2.33)	0.289
	high	46	0.97 (0.54, 1.75)	0.925
	extremely_high	41	0.38 (0.14, 1.01)	0.052

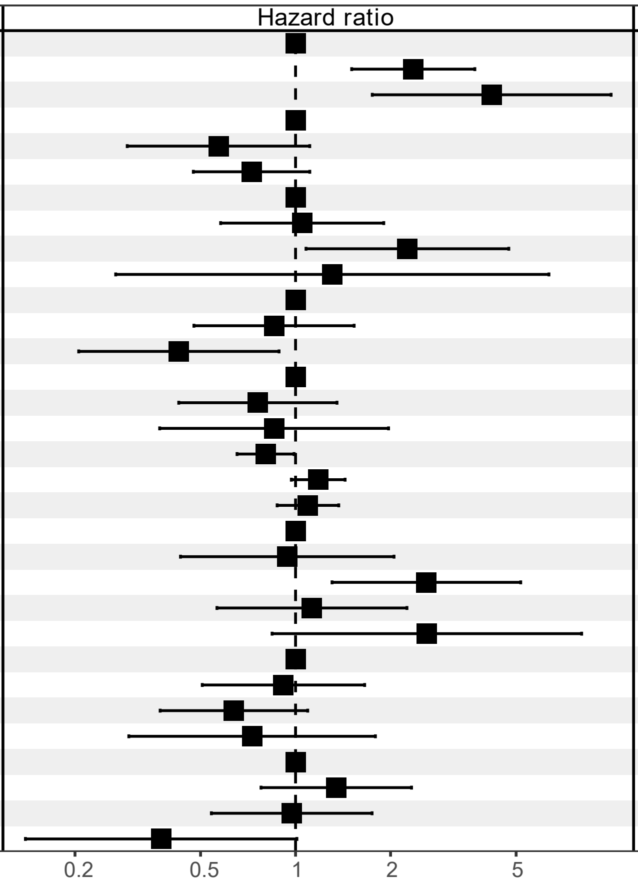

Supplementary Figure 6
